# Evaluating Syndromic surveillance systems at institutions of higher education (IHEs): A retrospective analysis of the 2009 H1N1 influenza pandemic at two universities

**DOI:** 10.1186/1471-2458-11-591

**Published:** 2011-07-26

**Authors:** Ying Zhang, Larissa May, Michael A Stoto

**Affiliations:** 1 Department of Health Systems Administration, Georgetown University, Washington, DC, USA; 2 Department of Emergency Medicine and Epidemiology and Biostatistics, The George Washington University, Washington, DC, USA

## Abstract

**Background:**

Syndromic surveillance has been widely adopted as a real-time monitoring tool for timely response to disease outbreaks. During the second wave of the pH1N1 pandemic in Fall 2009, two major universities in Washington, DC collected data that were potentially indicative of influenza-like illness (ILI) cases in students and staff. In this study, our objectives were three-fold. The primary goal of this study was to characterize the impact of pH1N1 on the campuses as clearly as possible given the data available and their likely biases. In addition, we sought to evaluate the strengths and weaknesses of the data series themselves, in order to inform these two universities and other institutions of higher education (IHEs) about real-time surveillance systems that are likely to provide the most utility in future outbreaks (at least to the extent that it is possible to generalize from this analysis).

**Methods:**

We collected a wide variety of data that covered both student ILI cases reported to medical and non-medical staff, employee absenteeism, and hygiene supply distribution records (from University A only). Communication data were retrieved from university broadcasts, university preparedness websites, and H1N1-related on campus media reports. Regional data based on the Centers for Disease Control and Prevention Outpatient Influenza-like Illness Surveillance Network (CDC ILINet) surveillance network, American College Health Association (ACHA) pandemic influenza surveillance data, and local Google Flu Trends were used as external data sets. We employed a "triangulation" approach for data analysis in which multiple contemporary data sources are compared to identify time patterns that are likely to reflect biases as well as those that are more likely to be indicative of actual infection rates.

**Results:**

Medical personnel observed an early peak at both universities immediately after school began in early September and a second peak in early November; only the second peak corresponded to patterns in the community at large. Self-reported illness to university deans' offices was also relatively increased during mid-term exam weeks. The overall volume of pH1N1-related communication messages similarly peaked twice, corresponding to the two peaks of student ILI cases.

**Conclusions:**

During the 2009 H1N1 pandemic, both University A and B experienced a peak number of ILI cases at the beginning of the Fall term. This pattern, seen in surveillance systems at these universities and to a lesser extent in data from other IHEs, most likely resulted from students bringing the virus back to campus from their home states coupled with a sudden increase in population density in dormitories and lecture halls. Through comparison of data from different syndromic surveillance data streams, paying attention to the likely biases in each over time, we have determined, at least in the case of the pH1N1 pandemic, that student health center data more accurately depicted disease transmission on campus at both universities during the Fall 2009 pandemic than other available data sources.

## Background

In the spring of 2009, a novel H1N1 influenza virus, now denoted pH1N1, emerged in North America and spread to the rest of the world in less than two months [1]. Soon afterwards, it became apparent that children and young adults were particularly vulnerable to infection [2-4]. This demographic pattern posed unique challenges for institutions of higher education (IHEs), which predominantly serve young adults. Moreover, some large residential universities operate as nearly self-sufficient communities in which the homogeneity of the campus population increases vulnerability to infectious diseases that target young adults, and transmission in universities can thus catalyze community-wide transmission [5].

Concerned with the re-emergence of the virus when students returned at the end of the summer, IHEs realized the need for surveillance systems capable of providing real-time situational awareness to guide the implementation of preventive measures to protect students' health and contingency plans to maintain basic educational functions. During the pH1N1 pandemic, the President's Council of Advisor on Science and Technology (PCAST) was recommending using syndromic surveillance, which, by using pre-diagnostic data, is thought to have a distinct advantage over the traditional surveillance method in terms of timeliness [6]. In response, the American College Health Association (ACHA) initiated a system to gather such data from IHEs and publish it weekly [7]. Surveillance of school absenteeism and other syndromic surveillance methods were used during pH1n1 outbreak in other countries as well [8,9].

The validity and utility of syndromic surveillance, however, or of particular types of syndromic data, are not well understood [10]. In order to assess its validity in IHE settings, we compared data from two major universities in Washington, DC that collected a variety of data that were potentially indicative of influenza-like illness (ILI) in students, faculty, and staff with each other and with external data. University A compiled and reviewed these data in real time to monitor and inform the university's response to the H1N1 pandemic, while University B served as a comparison for this analysis. The primary goal of this analysis was to characterize the impact of pH1N1 on the campuses as clearly as possible given the data available and their likely biases. In addition, we sought to evaluate the strengths and weaknesses of the data series themselves, in order to inform these two universities and other IHEs about real-time surveillance systems that are likely to provide the most utility in future outbreaks (at least to the extent that it is possible to generalize from this analysis).

## Methods

### Data Collection

We collected a wide a variety of data that covered both student influenza-like illness (ILI) cases reported to student health center (SHC) and hospital emergency department (ED) visits at both universities, and student ILI cases reported to non-medical staff, employee absenteeism, and hygiene supplies distribution records from University A. Unless otherwise noted below, all data series were available on a weekly basis. The sources are described in detail in Additional File 1, and summarized in table in Additional File 2.

#### The information environment

University broadcasts, preparedness website updates, and H1N1-related on-campus media reports were retrieved from emails, web pages and paper prints available on campus. H1N1-related messages were classified into five major categories for university A, which included information about the advice line, presence of flu cases, vaccination, instructions on voluntary reporting to deans and the availability of personal hygiene supplies. For university B, seven categories were available, including student health center data, hospital emergency department visits, pH1N1 hotline calls, vaccine and personal hygiene supplies, as well as the requirement for a physician's note for excused absences due to illness. All messages were counted based on their appearance in any of the media sources outlined above. In addition, relevant policies were collected and reviewed by interviewing key staff members.

#### Student ILI cases reported to medical personnel

The total number of ILI visits to the student health service and telephone consultations were collected using the following case definition: fever (> 100 F) AND (cough and/or sore throat) in the absence of a known cause other than influenza. Student identification data were reviewed by SHC staff to ensure that individuals were counted only once. Data were available on a daily basis from August 29, 2009 to April 30, 2010, and were aggregated by adding cases in each 7-day period from Saturday to the following Friday.

The number of clinic visits of ILI patients aged 17-24 years old at the EDs of hospitals associated with both universities was obtained from the ED electronic health records in the aggregate (number of cases/week). University A's ED data were retrieved based on the following criteria: age 17-24 years and fever, with other causes of fever than influenza manually filtered out. University B's ED visits for ILI were counted using following criteria: age 17-24 years old and a chief complaint of "flu" or "fever," or a discharge diagnosis of "influenza" or "viral syndrome." Student status was not available from either university ED. Data were available on a daily basis from August 29, 2009 to April 30, 2010, and were aggregated by adding cases in each 7-day period from Saturday to the following Friday.

#### Student ILI cases reported to non-medical personnel (University A only)

The number reported includes ILI cases in student athletes (as reported by their team trainers), student ILI cases self-reported to the deans of all four undergraduate schools, and ILI cases reported by resident assistants. Data other than deans' reports were available on a weekly basis from August 29, 2009 to April 30, 2010. Deans' reports were not available from all deans until September 12, 2009.

#### Employee absenteeism data (University A only)

Employee absenteeism data include real-time reports on ILI-related absences among Facilities Office and Dining Services staff, and employee absenteeism from 2009 and 2008, retrieved retrospectively from a payroll system that tracks employee absences for compensation purposes. The closest available data for non-union employees' were "unscheduled leave" days, whereas for unionized employees it was "sick leave." In order to simplify the analysis, the two data sources were added together with the awareness that the ILI-related absenteeism for both groups of employees may have been overestimated. No faculty members or student workers are represented in this dataset.

#### Supply distribution data (University A only)

Supply distribution data include the aggregate number of pre-packaged meals, masks and thermometers picked up in student resident halls, based on reports from the residence hall offices (RHO). The data were available from August 28, 2009 to April 10, 2010 on weekly basis.

#### External Data

For comparison purposes we used data based on the Centers for Disease Control and Prevention's (CDC) ILINet surveillance data [11], which reflect the proportion of outpatients visits that were for ILI, the American College Health Association (ACHA) data (labelled "attack rate" in ACHA sources) [12], and Google Flu Trends data (web queries) for Washington, DC [13]. Both the ILINet and ACHA data were available nationally and for Region 3 (Delaware, the District of Columbia, Maryland, Pennsylvania, Virginia, and West Virginia).

### Data Preparation

For the ACHA data series, the "attack rate" is defined by the ACHA as the number of weekly reports of new cases divided by the number of students in the IHEs' reports that week for each state, which were grouped according to CDC's regional categories (note that this is not the standard epidemiological definition). ILI cases and the school population of University A and B were removed from the ACHA region 3 data to minimize the influence on this external data stream from the study population.

To make the data from different sources comparable, all data, including the ACHA data (ILI attack rate), CDC ILINet data (percentage of hospital visits with ILI), and Google Flu Trends data (influenza related web queries) were normalized into an activity index by dividing the actual count in each week by the average count for that data series for the period from August 28 through December 18, 2009, a period for which data were available for all series and reflected the height of the Fall 2009 wave of pH1N1. This analysis is intended to identify the timing of the outbreak on each campus, not the absolute level of cases. In this analysis, we have made the assumption that the number of students is constant throughout the semester, at least relative to the fluctuation in the number of cases.

### Data Analysis

Because there are no data that describe the actual rates of pH1N1 infection, or its consequences, on the two campuses or the community in which they sit, we adopted a "triangulation" approach in which multiple contemporary data sources, each with different expected biases, are compared to identify time patterns that are likely to reflect biases versus those that are more likely to be indicative of actual infection rates. This public health systems research approach is grounded in the understanding that surveillance data are the result of decisions made by patients, health care providers, and public health professionals about health-care seeking behaviour and provision of health care and reporting suspected or confirmed cases to health authorities. Moreover, every element of this decision-making is influenced by the informational environment (i.e. media coverage, implementation of active surveillance), processing and reacting to the information on an individual level (i.e. the health care seeker's self-assessment of risk, incentives for seeking medical attention and self-isolation, the health care provider's ordering of laboratory tests), and technical barriers (i.e. communication infrastructure for data exchange, laboratory capacity), all of which change constantly.

### IRB approval

One of the authors (YZ) had access to some identified data in her efforts to compile data for operational purposes at University A, but all of the analyses for this paper were conducted with aggregate data only, and this research was treated as "exempt" by the IRBs of both universities.

## Results

**Figure 1:**
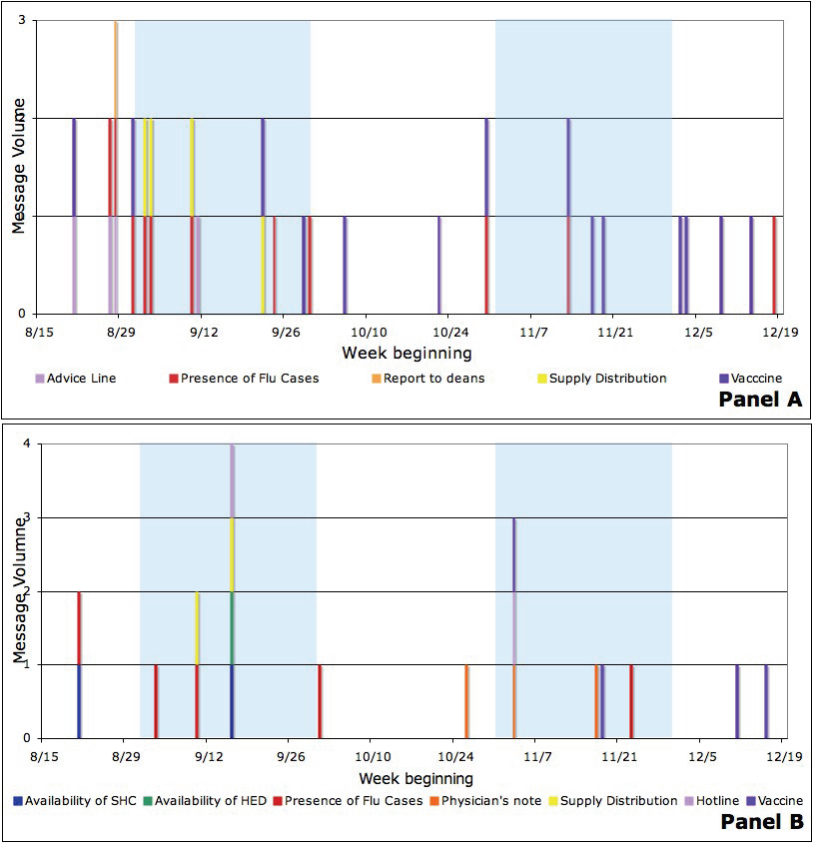
**Categorical pH1N1-related message timing and volume**. Panel a: University A. Panel b: University B.

In Panel A of Figure 1, the pH1N1 related messages reached its peak volume in late August and early September at University A, among which six out of sixteen messages were the situation-update announcements of the flu cases either in the region or on campus. Other messages included the instruction on utilizing on-campus resources, such as the H1N1 advice line and accessing personal hygiene supplies, as well as the recommendation for self-reporting of influenza-like-illness to deans. Later in November, there was another cluster of communication messages providing a situation update of flu cases on campus and the availability of H1N1 vaccination, while the volume was much lower compared to early September.

For University B, as shown in Panel B of Figure 1, communication messages were also clustered in early September and November. Four situation updates of flu cases were released during the first peak, together with instructions on accessing the student health center, visiting the university hospital emergency department, the H1N1 hotline and provision and use of personal hygiene supplies. Due to the policy change regarding excused absences, three messages were sent out requiring medical documentation to support any ILI related absence during mid-terms and finals.

**Figure 2:**
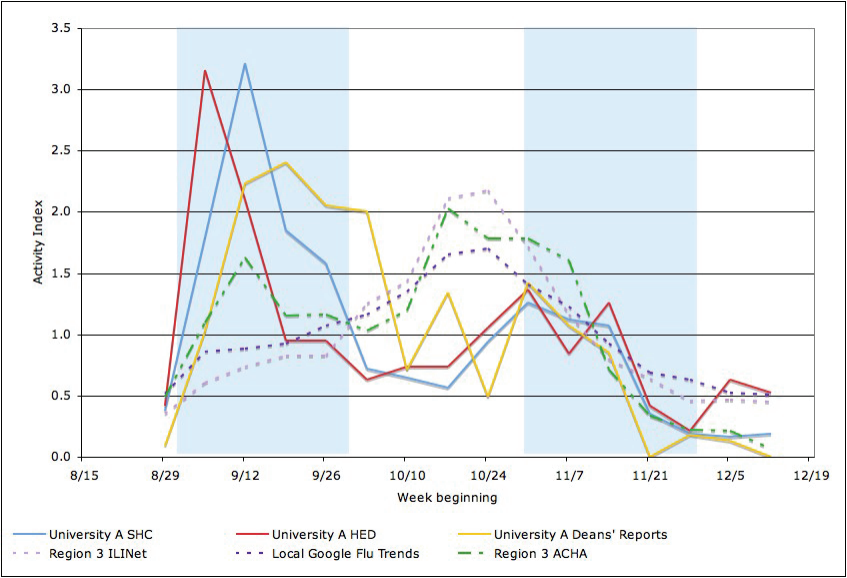
**University A student influenza-like illness (ILI) cases reported to student health center (SHC), hospital emergency department (HED) and deans' offices compared to regional American College Health Association (ACHA) influenza surveillance, CDC Outpatient Influenza-like Illness Surveillance Network (ILINet) and local Google Flu Trends data**.

As shown in Figure 2, there were two peaks in the 2009 Fall Semester at University A, the larger one in early September as students returned from summer vacation and the other in late October to early November. Reports to undergraduate deans were elevated relative to the other data series in October (i.e. they did not fall as sharply between the two peaks), which is the mid-term exam period. Figure 2 also demonstrates that this pattern differs from the ACHA ILI surveillance network data for Region 3, which exhibits a larger peak in late October than in September, and from the CDC ILINet for Region 3 and the Google Flu Trends data for Washington, DC, both of which show only one peak in late October.

**Figure 3:**
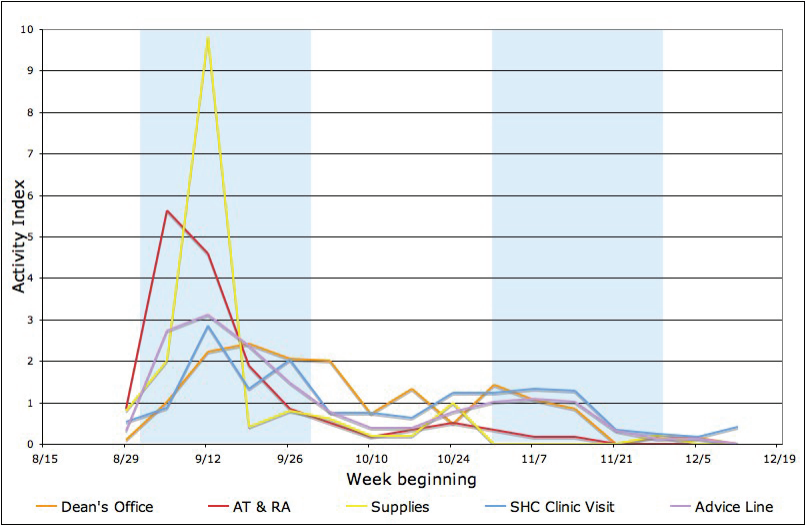
**Influenza-like illness (ILI) case reports to athletic trainers (AT) and resident assistants (RA), residence hall offices (RHO) requests for supplies, and calls to the H1N1 advice line compared to student health center (SHC) visits and dean's office reports, University A only**.

Figure 3, for University A only, compares the number of ILI case reports to athletic trainers (AT) and resident assistants (RA), RHO requests for supplies, and calls to the H1N1 advice line compared to SHC visits and dean's office reports. Relative to the other series, both AT and RA reports and RHO supply requests peaked earlier in September and are relatively higher than other series, perhaps reflecting the higher volume of messages (shown in Figure 1a) in September.

**Figure 4:**
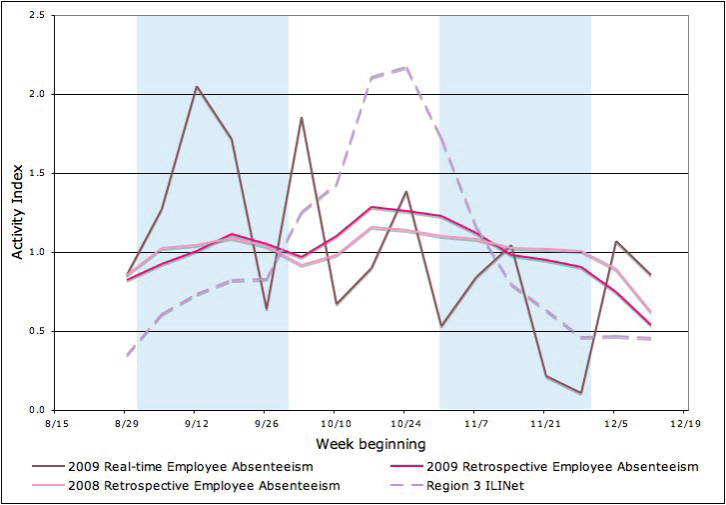
**Real-time and retrospective employee absenteeism, University A only**.

University A's employee absenteeism data are shown in Figure 4. Neither the real-time ILI-related employee absenteeism data nor retrospective data from the payroll systems exhibited any peaks in the Fall of 2009, suggesting that employees, who by and large are older than students, were apparently not as affected by pH1N1.

**Figure 5:**
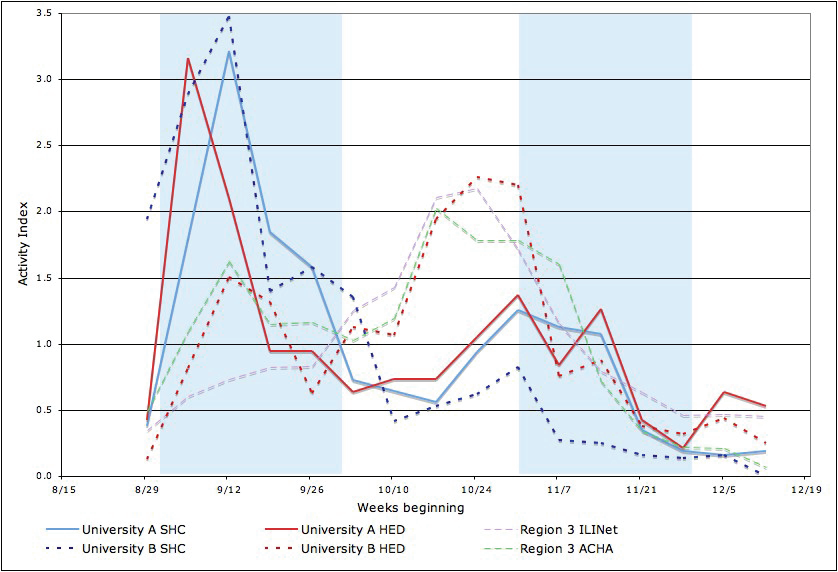
**Influenza-like illness (ILI) cases reported to student health centers (SHC) and hospital emergency departments (HED) from both universities compared to regional CDC Outpatient Influenza-like Illness Surveillance Network (ILINet) data**.

Figure 5 compares the data SHC and ED data from both universities, with the ACHA and ILINet data for Region 3 as a reference. The SHC data for the two universities are comparable. However, the ED data are not: University B's ED data exhibit a larger second peak in October than University A's ED data. University B's ED peak appears consistent with ILI activity in the larger community. Of note, in University B, the first peak of ILI activity is seen primarily in SHC visits, while the second peak is seen in ED visits.

## Discussion

The primary limitation of this analysis is the lack of definitive knowledge about the actual number of pH1N1 cases at the two universities - a "gold standard." To address this problem we developed an approach that compares ("triangulates") multiple data systems, each with its own expected biases over time, to identify those that most likely mirror actual disease trends. Epidemiologists are typically aware of these potential biases in a qualitative sense, and present their analysis of the available data with appropriate caveats. In our approach, which benefits from hindsight, we attempt to use information about the likely direction and time patterns of these biases to understand the surveillance system and the validity and utility of different syndromic surveillance data sources. This type of analysis is necessarily qualitative and contextual; rather than serving as a recipe for doing this in other settings, this analysis should be seen as an example that illustrates the concept. This analysis also illustrates to the call in U.S. National Health Security Strategy Implementation Plan (released for public comment in 2010) for the development, refinement, and wide-spread implementation of quality improvement tools, specifically methods "to collect data ... from real incidents ... to identify gaps, [and] recommend and apply programs to mitigate those gaps [14]."

Another limitation of the data analysis is the uncertainty of whether the ILI cases captured by the surveillance system were pH1N1. As recommended by the CDC interim guidelines [15], both universities stopped routinely testing for pH1N1 in early September. Although the CDC Virologic Surveillance data for region 3 suggested that the predominant proportion of the test positive specimens were subtyped as pH1N1, the total percentage for test positive samples varied from 4.4% to 55.9% in weeks 35 to 50 [16]. Because this proportion varies so much, trends in reported ILI cases may not reflect trends in actual H1N1 infection.

As described in more detail below, this approach suggests that the peak in cases at both universities at the beginning of the semester, a peak not seen in data for the surrounding community, is probably real and a reflection of expected disease dynamics. The lower peak, especially at University A, when pH1N1 was widespread in the community might reflect the removal of susceptible cases earlier in the semester, or simply surveillance fatigue. This analysis also suggests surveillance artifacts - surveillance fatigue and changing incentives driven by the exam schedule - that are likely to influence surveillance data in future outbreaks, and that should be taken into account in the interpretation of these data.

### Unique transmission pattern in IHEs

Both universities experienced the first and the highest peak in student ILI cases immediately after Fall semester classes started in early September 2009, which corresponds to peaks found in other universities and colleges in Region 3 (Delaware, the District of Columbia, Maryland, Pennsylvania, Virginia, and West Virginia). It should be noted, however, that both of these universities contributed to the ACHA reports. The CDC ILINet data for the same region and Google Flu Trends data for Washington, DC, on the other hand, did not peak until late October. University A also experienced a second, lower peak in cases with a two-week delay in early November, according to the SHC and ED data. When comparing the SHC and ED data from University A and University B (as shown in Figure 5), University B's ED data differ from other data sets from both universities, and show a transmission pattern that resembles the CDC ILINet data. Since the ILI cases from both hospital EDs are not restricted to students, they include not only student cases but also other 17 to 24 year old adults in the community and from other parts of the city. Unlike University A which is not inaccessible by mass transit, University B is located in a part of Washington that has a large population of young adults and easy access to the public transportation, so the higher volume of young adult visits at University B ED during November, when the virus was circulating in the general population, may not have come from the college population.

In the comparison between ACHA and CDC ILINet data across all states, the tendency of an early increase in ILI cases among college students in seven out of ten regions, as shown in Additional File 3. Together with our findings, this suggests that the difference in the timing of peaks reflects the differences between college students and the general public. This is plausible given that students are in an age group at higher risk for infection. Moreover, students returning to campus for the Fall term may have carried the virus from their home states, and the sudden increase of population density in dormitories and lecture halls may also have contributed to the rapid onset of the outbreak on campus due to facilitated transmission. Thus, it seems likely that on a national level, residential IHEs tended to experience an early peak immediately after the Fall term began in 2009.

### Influence of incentives and informational environment

All of the data analysed in this report are based, to some degree, on students and staff taking action based their illness. Such behaviour is driven not only by the fact of being sick, but also by the incentives to report, including perceptions of barriers to help-seeking behaviour (i.e. geographic distance, queuing, chance of exposure to other infected patients), the likely benefit to be gained (medical and non-medical) by reporting, the timeliness of the help to be delivered, as well as the informational environment the students and staff are exposed to. In particular, two factors - surveillance fatigue and reporting incentives - seem capable of explaining some of the patterns in the data.

#### Surveillance fatigue

As seen in Figure 1, the Fall semester at both universities began with a high awareness of pH1N1. At University A, a number of new *ad hoc *surveillance systems were developed, some of which required a substantial reporting burden by students and staff at the student health center and deans' offices, athletic trainers and RAs, and of course the students themselves. Over the course of the semester, however, it became apparent that although pH1N1 was widespread in children and young adults, it was not as virulent as feared [17,18], and the frequency of H1N1 messages dropped. It would not be surprising, therefore, that staff who put a substantial effort into reporting ILI cases in September were less enthusiastic about it as the semester wore on, and possibly less complete in their reporting. Moreover, since most students who presented themselves for medical attention early in the semester did not receive anti-viral or other specific treatment as per the CDC guidelines, it seems likely that their friends and roommates who became ill later in the semester saw no reason to seek medical care.

Surveillance fatigue is likely to be more obvious in systems that use human resources not primarily designated for disease prevention and health promotion. For instance, the reports from the RA at University A increased to their highest level in the first week after classes resumed and dropped dramatically afterwards. Although ILI activity could still be observed from other data sources after the second peak through Spring 2010, the reports from RAs completely stopped at the end of November. The RA reporting system might have been sensitive to student ILI cases in the early stages, considering the relatively low barrier of utilizing the resources (close proximity, no queuing), and the expectation of immediate help (supply distribution, accommodation relocation). However, when the reporters and those receiving the reports are all laypersons to public health practice, fading interest can be magnified in the microenvironment between the two parties.

#### Reporting incentives

At University A, undergraduate students were instructed to notify their deans about their influenza-like illness as a substitute for medical proof of illness otherwise required to justify absence from class. This was published on August 28, 2009, and not emphasized afterwards. However, as noted in Figure 2, reports to undergraduate deans at University A were elevated relative to the other series in October, when mid-term exams were scheduled in many classes. Thus it seems likely that the relative number of reports to deans at University A during this period (or more precisely the failure for the number to drop as sharply as other data series), may reflect the increased need for students to have medical excuses for exams rather than for ordinary classes, where attendance is usually not taken.

### Evaluation of syndromic surveillance data systems

To translate these results into recommendations for IHEs regarding the design and implementation of surveillance systems for future disease outbreaks, other factors must also be taken into account. For instance, surveillance activities conducted by trained health care workers are more likely to capture actual ILI cases based on clinical findings. Moreover, well-informed healthcare workers who conduct surveillance as part of their regular responsibilities are more likely to maintain a relatively stable and predictable report triggering threshold, in line with the CDC and WHO (World Health Organization) guidelines [19-21]. On the other hand, *ad hoc *reporting systems may be more sensitive to changes in the informational environment. When the reporting channels are relatively new, communication messages designed to encourage their use might have a short-term effect when they are released, but surveillance fatigue may set in quickly when the intensity of media coverage decreases and public interest fades. In addition, the expected benefits of presenting oneself to the reporting system, and how easily reports can be made, may also have an impact on the direction and scope of the bias. In a pandemic characterized by low virulence and limited treatment options for young adults, the expected benefits of seeking care decreased over time, except for the mid-term exam effect observed in Deans' reports at University A. Thus, this assessment suggests that at least for outbreaks similar to pH1N1, student health center data, though biased by surveillance fatigue, provides the most accurate and useful data.

## Conclusions

During the 2009 H1N1 pandemic, University A and B both experienced a peak number of ILI cases at the beginning of the Fall term. This pattern, seen in a variety of surveillance systems at these universities and to a lesser extent in data from other IHEs, most likely results from students bringing the virus back to campus from their home states coupled with a sudden increase in population density in dormitories and lecture halls.

Through comparison of data from different syndromic surveillance data streams, paying attention to the likely biases in each over time, we have determined, at least in the case of the pH1N1 pandemic, that student health center data more accurately depicted transmission on campus in both universities during the Fall 2009 pandemic than other available data sources. Although maintaining an unduplicated list from visits and phone calls was time consuming, it was felt to be necessary to manage the situation. Other systems that were used at University A required major staff efforts to collect the data and were apparently less accurate. Reporting systems based on student reports to their deans may be relatively inflated during examination periods or other times when it students need to be formally excused from class, but such systems combined with a liberal excused absence policy (not requiring a physician's note) can help to relieve over-utilization of medical resources for non-medical purposes.

## Competing interests

The authors declare that they have no competing interests.

## Authors' contributions

YZ and LM collected data from University A and B, respectively. All authors participated in designing the study, analysing and interpreting the data and drafting the manuscript. All authors read and approved the final manuscript.

## Pre-publication history

The pre-publication history for this paper can be accessed here:

http://www.biomedcentral.com/1471-2458/11/591/prepub

## Supplementary Material

Additional file 1 **Data sources**. Detailed description of data sources, including case definitions, collection protocols, and available dates. Click here for file

Additional file 2 **Summary surveillance systems**. A summary table comparing surveillance systems analysed in terms of case definitions, populations covered, reporter, collection methods, and timeliness. Click here for file

Additional file 3 **Influenza activity in the IHE and general populations**. Comparison of American College Health Association (ACHA) influenza surveillance attack rate data for the institutions of higher education (IHE) population and CDC Outpatient Influenza-like Illness Surveillance Network (ILINet) influenza-like illness for the general population, by region, United States, August 22 through December 12, 2009. Click here for file

## References

[B1] World Health Organization (WHO) Pandemic (H1N1) 2009 - update 103 http://www.who.int/csr/don/2010_06_04/en/index.html

[B2] World Health Organization (WHO) Global Alert and Response (GAR): Influenza-like illness in the United States and Mexico http://www.who.int/csr/don/2009_04_24/en/

[B3] Centers for Disease Control and Prevention (CDC) Patients hospitalized with 2009 pandemic influenza A (H1N1) - New York City, May 2009 MMWR Morb Mortal Wkly Rep 2010 58 (51):1436 1440 20057350

[B4] Echevarria-Zuno S Mejia-Arangure JM Mar-Obeso AJ Grajales-Muniz C Robles-Perez E Gonzalez-Leon M Ortega-Alvarez MC Gonzalez-Bonilla C Rascon-Pacheco RA Borja-Aburto VH Infection and death from influenza A H1N1 virus in Mexico: A retrospective analysis Lancet 2009 374 9707 2072 2079 19913290 10.1016/S0140-6736(09)61638-X

[B5] Chao DL Elizabeth Halloran M Longini IM Jr School opening dates predict pandemic influenza A(H1N1) outbreaks in the United States J Infect Dis 2010 202 6 877 880 10.1086/655810 20704486 PMC2939723

[B6] President's Council of Advisors on Science and Technology (PCAST) Report to the President on U.S. Preparation for 2009-H1N1 Influenza 2009 25

[B7] ACHA American College Health Association influenza-like illnesses (ILI) surveillance in colleges and universities 2009-2010: Weekly college ILI cases reported http://www.acha.org/ILI_Project/ILI_Surveillance.cfm

[B8] Schmidt WP Pebody R Mangtani P School absence data for influenza surveillance: A pilot study in the United Kingdom Euro Surveill 2010 15 3 19467 20122378

[B9] Sigmundsdottir G Gudnason T Olafsson O Baldvinsdottir GE Atladottir A Love A Danon L Briem H Surveillance of influenza in Iceland during the 2009 pandemic Euro Surveill 2010 15 49 19742 21163181 10.2807/ese.15.49.19742-en

[B10] Stoto MA Syndromic surveillance in public health practice Institute of Medicine, ed. Infectious Disease Surveillance and Detection (Workshop Report) 2007 Washington: National Academy Press 63 72

[B11] Centers for Disease Control and Prevention (CDC) Weekly percent of visits for influenza-like Illness (ILI) reported by the U.S. Outpatient Influenza-like Illness Surveillance Network (ILINet) summary for HHS region 3 (DE,DC,MD,PA,VA,WV) http://www.cdc.gov/flu/weekly/regions2009-2010/senreg3.htm

[B12] ACHA American College Health Association Influenza Like Illnesses (ILI) surveillance in colleges and universities 2009-2010: Weekly college ILI cases reported http://www.acha.org/ILI_Project/ILI_Surveillance.cfm

[B13] Google.org Flu Trends Explore Flu Trends - United States http://www.google.org/flutrends/us/#US-DC

[B14] U.S. Department of Health and Human Services Interim implementation guide for the national health security strategy of the United States of America. 2009

[B15] Centers for Disease Control and Prevention (CDC) Interim recommendations for clinical use of influenza diagnostic tests during the 2009-10 influenza season http://www.cdc.gov/h1n1flu/guidance/diagnostic_tests.htm

[B16] Centers for Disease Control and Prevention (CDC) Influenza isolates from HHS region 3 reported by WHO/NREVSS collaborating laboratories 2009-2010 season http://www.cdc.gov/flu/weekly/regions2009-2010/data/whoreg3t.htm

[B17] World Health Organization (WHO) WHO Global Alert and Response (GAR) - Clinical features of severe cases of pandemic influenza http://www.who.int/csr/disease/swineflu/notes/h1n1_clinical_features_20091016/en/index.html

[B18] Presanis AM De Angelis D New York City Swine Flu Investigation Team Hagy A Reed C Riley S Cooper BS Finelli L Biedrzycki P Lipsitch M The severity of pandemic H1N1 influenza in the United States, from April to July 2009: A Bayesian analysis PLoS Med 2009 6 12 e1000207 10.1371/journal.pmed.1000207 19997612 PMC2784967

[B19] Centers for Disease Control and Prevention (CDC) Interim recommendations for clinical use of influenza diagnostic tests during the 2009-10 influenza season http://www.cdc.gov/h1n1flu/guidance/diagnostic_tests.htm

[B20] Centers for Disease Control and Prevention (CDC) Interim guidance for state and local health departments for reporting influenza-associated hospitalizations and deaths for the 2009-2010 season http://www.cdc.gov/H1N1flu/hospitalreporting.htm

[B21] World Health Organization (WHO) Human infection with pandemic (H1N1) 2009 virus: Updated interim WHO guidance on global surveillance http://www.who.int/entity/csr/disease/swineflu/guidance/surveillance/WHO_case_definition_swine_flu_2009_04_29.pdf

