# Supplementary material for: Evaluating Syndromic surveillance systems at institutions of higher education (IHEs): A retrospective analysis of the 2009 H1N1 influenza pandemic at two universities
Source: BMC Public Health. 2011 Jul 26;11:591. doi: 10.1186/1471-2458-11-591 (PMC3151236; doi:10.1186/1471-2458-11-591)
Supplement: Additional file 1 — Data sources. Detailed description of data sources, including case definitions, collection protocols, and available dates. [file 1471-2458-11-591-S1.DOC]

**Data Source Detail**

The following paragraphs provide detailed information on the data sources and collection procedures.

- **Communications data**
  - University broadcasts, preparedness website updates, and H1N1-related on campus media reports were retrieved from emails, web pages and paper prints available on campus. H1N1-related messages were classified into four major categories for university A and six for university B, and counted based on the appearance in any of the media sources outlined above. In addition, relevant policies were collected and reviewed by interviewing key staff members.
- **Student ILI cases reported to medical staff**
  - *University A hospital emergency department visits*. University A hospital emergency room visits records were retrieved based on the following searching criteria: age 17-24 years and fever. Fever cases with other obvious causes were manually filtered out. Student status was not known, so some may be other in that age range living in the community. These data are available on a daily basis from August 30th, 2009 to March 26th, 2010, and is aggregated by adding cases in each 7-day period starting at midnight on Saturday.
  - *University A student health center*. This data set includes the total of visits to the Student Health Center, off-hour calls to the Student Health Center, and calls to the nurse-operated H1N1 advice line. Those who met the following criteria were counted: fever (> 100F) AND (cough and/or sore throat) in the absence of a known cause other than influenza. Logs were reviewed to ensure that individuals were counted only once. These data are available on a daily basis from August 30th, 2009 to April 14th, 2010, and are aggregated by adding cases in each 7-day period starting at midnight on Saturday.
  - *University B hospital emergency department visits*. Data from the ED electronic health record (Picis, inc.) was obtained retrospectively in aggregate (number of cases/week). University B ED visits for ILI were included using following criteria: age 17-24 years old and the following: chief complaint of “flu” or “fever” or discharge diagnosis of “influenza” or “viral syndrome”. Student status was not available. Data were available on a daily basis from August 29th, 2009 to April 30th, 2010, and were aggregated by adding cases in each 7-day period.
  - *University B student health service*. This data set included the total number of ILI related patient visits to the student health service, and telephone consultations. Those who met the following CDC criteria were counted: fever (> 100F) AND (cough and/or sore throat) in the absence of a known cause other than influenza. These data correspond to that data submitted to the CDC, as a sentinel provider site for ILINet. The ILI cases report is available from the 35th week of 2009 till the 21st week of 2010; however University B SHS extended collection of data due to the H1N1 pandemic. These data were collected on a daily basis during the novel H1N1 pandemic period, from August 30th, 2009 to April 14th, 2010, and are aggregated by adding cases in each 7-day period starting at midnight on Saturday.
- **Student absenteeism reported to non-medical staff (University A**)
  - *ILI cases from deans*. Dean’s offices at the four undergraduate colleges collected ILI related student absence data based on the emails and phone calls from individual students. These data were collected on weekly basis, from Saturday to the following Friday. It was not until September 12th, 2009 that all four colleges participated in reporting. Data are available from August 29th, 2009 to March 5th, 2010.
  - *ILI cases reported by athletic trainers (AT) and resident assistants (RA)*. Athletic team trainers kept track of individual student athletes and report ILI cases with information regarding symptoms, date of symptom onset, duration, and the temperature taken by the head athletic trainer. The information was reported on weekly basis, from Saturday to Friday, available from August 29th, 2009 to March 5th, 2010. Resident assistants also collected reports from the sick students or their roommates on daily basis. Data were reviewed so that no student is counted twice in the same week. These data were aggregated by adding cases in each 7-day period starting at midnight on Saturday, and is available from August 29th, 2009 to March 5th, 2010, although no new cases were reported from RA after November 20th, 2009.
- **Employee absenteeism data (University A)**
  - *Real-time employee absenteeism*. The real-time employee absence data came from the call-in sick log at the Facilities Office and Dining Services. The Facilities Office log is available on daily basis from June 1, 2009 to May 28th, 2010, and is aggregated by adding cases in each 7-day period starting at midnight on Saturday. The timekeeper reviewed the log so that the individuals are counted once for leaves taken in consecutive days. The reason for sick leave can be any kind of physical discomfort. Employee call-in sick data from Dining Services are reported on weekly basis, from Saturday to the following Friday. Only ILI-related absence is recorded. These data are available from September 5th, 2009 to March 5th, 2010.
  - *Retrospective employee absenteeism of 2009 and 2008***.** This dataset is retrieved from the payroll system, which keeps tracking employee absences for management purposes. Non-union employees are paid on either bi-weekly or monthly basis and only recorded for “unscheduled leave,” while the employees represented by the union are paid on a bi-weekly basis and recorded as “sick leave.” In order to simplify and collapse the datasets, we add two groups of data together with the awareness that we may have been overestimating the ILI-related absenteeism for non-union employees. Also, since the starting and ending points are not constant in every week, to make it comparable to other datasets, we calculate the average incidence per day for every payroll cycle and calculate weekly absence incidence based on the cycle from Saturday to Friday. No faculty members, students, Facilities Office or Dining Services employees are represented in this dataset. These data is available from July 1st 2008 to December 31st 2009.
- **Supply distribution data (University A only)**
  - Supply distribution data includes the aggregate number of pre-packaged meals, masks and thermometers picked up in student resident halls, based on reports from the Residence Hall Offices (RHO). Data is available from August 28, 2009 to April 10, 2010 on weekly basis.
- **External Surveillance Data**
  - *ACHA*. American College Health Association Pandemic Influenza Surveillance Network collected reports on a voluntary basis from institutions of higher education regarding the number of new cases of ILI [ICD-CM Diagnosis 487.1]. Data reported are for the weekly period (Saturday through Friday), reported to ACHA as of the following Monday [1]. ACHA Regional Data refers to data for region 3 (DE, DC, MD, PA, VA, WV). These data are available from August 22nd, 2009 to April 30th, 2010. The attack rate is calculated based on the new cases and the number of students in the IHEs’ reports that week.
  - *ILINet.* The CDC Outpatient Influenza-like Illness Surveillance Network consists of weekly reports from more than 3,000 healthcare providers nationwide on the proportion of their patients with ILI (fever (> 100F) AND (cough and/or sore throat) in the absence of a known cause other than influenza. These data are available on CDC Flu View website on a weekly basis throughout the year, and the week runs from Saturday through the following Friday [2].
  - *Google Flu Trends.* The website Google Flu Trends, developed by Google.org, uses aggregated Google search data on influenza-like illness (ILI) symptoms to estimate flu activity “up to two weeks faster than traditional systems” [3]. The ILI related web queries data in Washington, DC area is available on its website and updated on daily basis. In previous studies, Google Flu Trends data shows high consistency with CDC surveillance data, and has been used as an estimate of CDC data on local level. Since the DC Google Flu Trends data corresponds well to region 3 CDC ILINet data in general pattern, we use regional CDC ILInet data as an estimate for A/H1N1 transmission in community in Washington, DC area.

**References**

1. [<http://www.acha.org/ILI_Project/ILI_Surveillance.cfm>]

2. [<http://www.cdc.gov/flu/weekly/fluactivity.htm>]

3. [[http://www.google.org/flutrends/us/#US-DC](http://www.google.org/flutrends/us/#US-DC)]
