# Supplementary material for: Evaluating Syndromic surveillance systems at institutions of higher education (IHEs): A retrospective analysis of the 2009 H1N1 influenza pandemic at two universities
Source: BMC Public Health. 2011 Jul 26;11:591. doi: 10.1186/1471-2458-11-591 (PMC3151236; doi:10.1186/1471-2458-11-591)
Supplement: Additional file 2 — Summary surveillance systems. A summary table comparing surveillance systems analysed in terms of case definitions, populations covered, reporter, collection methods, and timeliness. [file 1471-2458-11-591-S2.DOCX]

|  | Surveillance System | Case Definition | Population | Reporter | Timeliness | Active/Passive | Data Collection Methods |
| --- | --- | --- | --- | --- | --- | --- | --- |
| University A | SHC - Clinic Visit + off-hour log | Fever (> 100F) AND (cough and/or sore throat) in the absence of a known cause other than influenza | University A students | Clinicians | Daily | Passive | Manual |
|  | SHC - Hotline | Fever (> 100F) AND (cough and/or sore throat) in the absence of a known cause other than influenza | University A students | Nurses | Daily | Passive | Manual |
|  | Emergency Department Visits | Fever cases in the absence of a known cause other than influenza | ED Patients aged 17-24 years | Clinicians | Daily | Passive | Electronic |
|  | Deans’ Reports | Student self-reported influenza-like-illness via emails and phone calls | University A students | Deans | Weekly | Passive | Manual |
|  | Athletic Trainers’ Reports | Student self-reported influenza-like-illness, temperature taken by the head athletic trainer | University A students | Athletic Trainers | Weekly | Passive | Manual |
|  | Resident Assistants’ Reports | Student self-reported influenza-like-illness | University A students | Resident Assistants | Daily | Passive | Manual |
|  | Supply Distribution | Pre-packaged meals, masks and thermometers picked up in student resident halls | University A students | Resident Hall Offices Staff | Weekly | Passive | Manual |
|  | Real-time Employee Absenteeism | Employee self-reported influenza-like-illness via phone calls | University A Facilities Office and Dining Services employees | Timekeeper | Daily | Passive | Manual |
|  | Retrospective Employee Absenteeism | “Unscheduled leave” taken by non-union employees and “sick leave” taken by employees from the union. | University A employees (excluding faculty and employees from Facilities Office and Dining Services) | Human Resources Staff | Bi-weekly/ Monthly | Passive | Electronic |
| University B | SHS: Clinic visits + telephone consultations | Fever (> 100F) AND (cough and/or sore throat) in the absence of a known cause other than influenza | University B students | Clinicians | Daily | Passive | Manual |
|  | Emergency Department visits | Chief complaint of “flu” or “fever” or discharge diagnosis of “influenza” or “viral syndrome”. | ED Patients aged 17-24 years | Clinicians | Daily | Passive | Electronic |
| External Data | ACHA | Fever (> 100F) AND (cough and/or sore throat) in the absence of a known cause other than influenza | Students at participating universities and colleges | Clinicians | Weekly | Passive | Electronic |
|  | ILINet | ILI (fever (> 100F) AND (cough and/or sore throat) in the absence of a known cause other than influenza | General public | Clinicians | Weekly | Passive | Electronic |
|  | Google Flu Trends | Influenza related web queries | General public | Google searching engine | Daily | Passive | Electronic |
