# Supplementary figures and images for: Evaluating Syndromic surveillance systems at institutions of higher education (IHEs): A retrospective analysis of the 2009 H1N1 influenza pandemic at two universities
Source: BMC Public Health. 2011 Jul 26;11:591. doi: 10.1186/1471-2458-11-591 (PMC3151236; doi:10.1186/1471-2458-11-591)

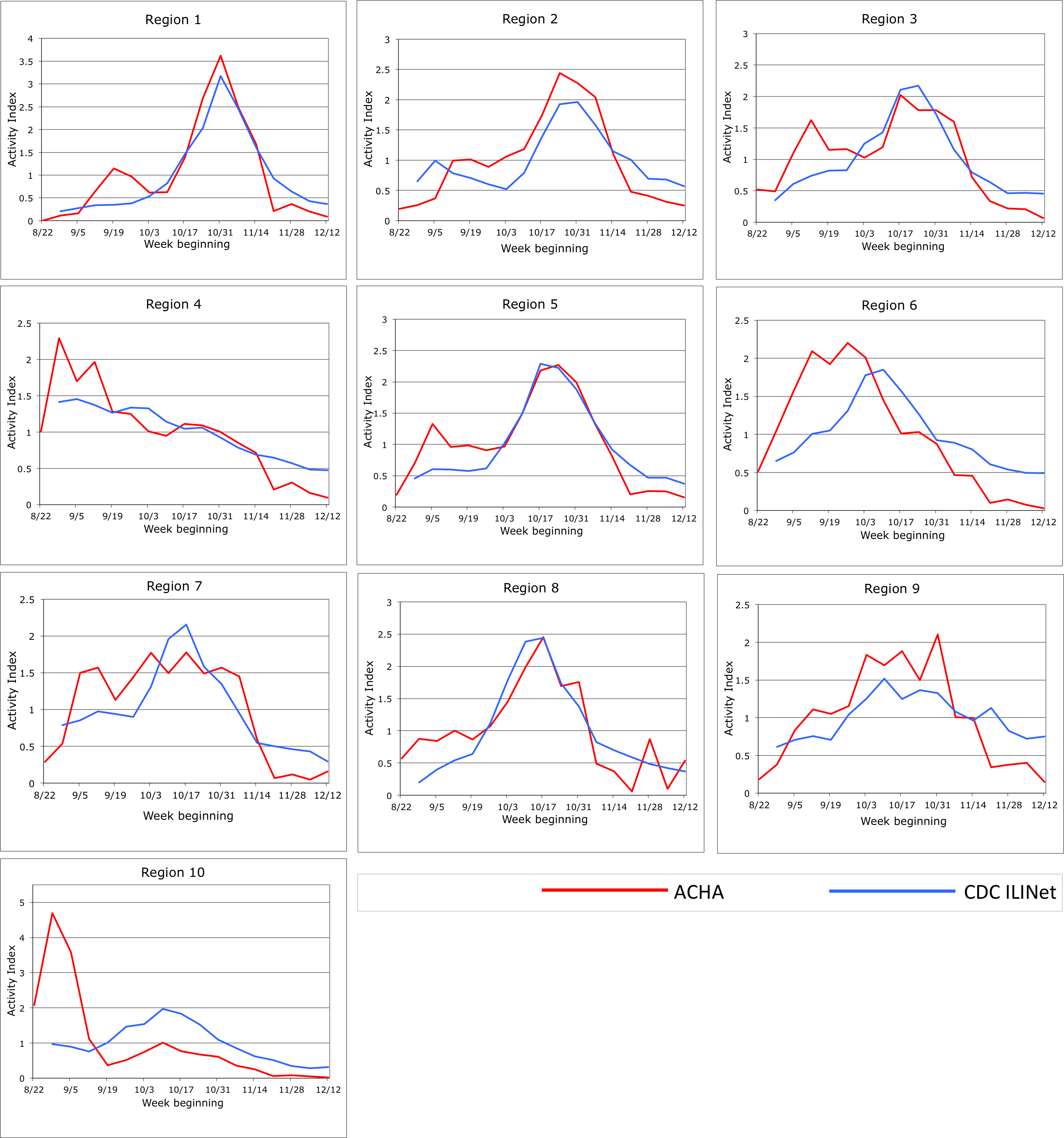

Supplement: Additional file 3 — Influenza activity in the IHE and general populations. Comparison of American College Health Association (ACHA) influenza surveillance attack rate data for the institutions of higher education (IHE) population and CDC Outpatient Influenza-like Illness Surveillance Network (ILINet) influenza-like illness for the general population, by region, United States, August 22 through December 12, 2009. [file 1471-2458-11-591-S3.PNG]
